# Special Considerations for Multiple Limb Amputation

**DOI:** 10.1007/s40141-014-0067-9

**Published:** 2014-10-24

**Authors:** Paul F. Pasquina, Matthew Miller, A. J. Carvalho, Michael Corcoran, James Vandersea, Elizabeth Johnson, Yin-Ting Chen

**Affiliations:** 1 Department of Physical Medicine & Rehabilitation, Uniformed Services University of the Health Sciences, 4301 Jones Bridge Road, Bethesda, MD 20814 USA; 2 Department of Rehabilitation, Walter Reed National Military Medical Center, Bethesda, MD USA; 3 Center for Rehabilitation Sciences Research (CRSR), Uniformed Services University of the Health Sciences, Bethesda, MD USA; 4 Henry M. Jackson Foundation for the Advancement of Military Medicine, Bethesda, MD USA; 5 Medical Orthotics and Prosthetics, Silver Spring, MD USA; 6 Advanced Arm Dynamics, Bethesda, MD USA

**Keywords:** Multiple limb loss, Multiple limb amputation, Prosthetics, Assistive technology, Pain management, Behavioral health, Rehabilitation, Amputation, Adaptive technology

## Abstract

It has been estimated that more than 1.6 million individuals in the United States have undergone at least one amputation. The literature abounds with research of the classifications of such injuries, their etiologies, epidemiologies, treatment regimens, average age of onset (average age of amputation), and much more. The subpopulation that is often overlooked in these evaluations, however, is comprised of individuals who have suffered multiple limb loss. The challenges faced by those with single-limb loss are amplified for those with multiple limb loss. Pain, lifestyle adjustment, and quality of life return are just a few key areas of concern in this population. Along with amputations resulting from trauma, many individuals with multiple amputations have endured them as a result of dysvascular disease. Over recent years, amputations as a result of dysvascular disease have risen to comprise more than 80 % of new amputations occurring in the United States every year. This compares to just 54 % of total current prevalence. Those with diabetes comorbid with dysvascular disease make up 74 % of those with dysvascular amputations, and these individuals with diabetes comorbid with dysvascular disease have a 55 % chance of enduring an amputation of their contralateral limb within 2–3 years of their initial amputation. With the well-documented aging of the nation’s population and the similarly skyrocketing prevalence of dysvascular disease and diabetes, it can be expected that the number of individuals with multiple limb loss will continue to increase in the United States. This article outlines the recommended measures of care for this particular subpopulation, including pain management, behavioral health considerations, strategies for rehabilitation for various levels and variations of multiple limb loss, and the assistive technology and adaptive equipment that might be available for these individuals to best enable them to continue healthy, fulfilling lives following amputation.

## Introduction

The prevalence of limb loss in the United States was estimated to be 1.6 million in 2005, and is anticipated to more than double by the year 2050, at which point it will affect nearly 1 in every 120 individuals [1•]. The most common causes of amputation include vascular disease, trauma, cancer, and congenital deformities. Vascular disease and trauma represent the vast majority of individuals with amputation, accounting for 54 and 45 %, respectively. Amputation, secondary to cancer or congenital deformities, occurs much less frequently; only a combined 2.5 % of the total population of individuals with limb loss attribute it to one of these two etiologies [2]. While exact numbers are not known, it is estimated that approximately 7.3 % of individuals with trauma-associated amputation have multiple limb loss [3]. During U.S. military operations in Afghanistan and Iraq, rates of multiple limb loss ranged from 20 to 60 % [4]. Unlike these patients, those with amputation resulting from vascular disease, amputation of one limb does not prevent the progression of disease of the contralateral limb [5]. Several studies have reported that within one year of initial amputation, 11.9–15 % of individuals with dysvascular disease will undergo contralateral limb amputation [6]. Within two years, the risk increases to 18–26 % and to 27–44 % within 4 years [7–9].

Approximately 133,235 amputation-related hospital discharges occur in the United States every year [10••]. Successive studies have shown that the distribution of trauma-related amputations has been decreasing since the 1980s to a low of 16 % of new cases according to some recent reports [2, 9, 11]. Cancer- and congenital disease-related amputations have seen similar drops to less than 1 % of all cases. In comparison, dysvascular amputations have risen to comprise 82 % of amputations [12]. The majority of individuals who undergo a dysvascular amputation are elderly and nearly 97 % of the amputations performed are of the lower limbs. The well-documented aging of the population, coupled with increasing rates of conditions such as peripheral vascular disease and diabetes, continue to contribute to these trends [13]. As the rates of vascular disease-related amputation increase, so too have the cases of multiple limb loss.

### Vascular Disease

Dysvascular amputations typically occur subsequent to the development of diabetes or peripheral arterial disease (PVD). Dillingham et al. found that just over 74 % of Medicare patients with dysvascular amputation also had diabetes [4]. It is likely that the co-existence of multiple comorbidities, such as diabetes, peripheral vascular disease, and renal disease, have a combined effect on the risk for amputation, contributing to the development of gangrene, sepsis, and other diseases that eventuality necessitate amputation.

Currently, 25.8 million people are impacted by diabetes in the United States and this number is expected to double by the year 2030 [1•, 14]. Individuals with diabetes have an approximate tenfold increase in risk of amputation than those without diabetes, and approximately 55 % of individuals who sustain an amputation secondary to vascular disease and diabetes will require an amputation of the contralateral limb within 2–3 years [15]. In addition, nearly half of people who have an amputation because of vascular disease will die within 5 years [16].

### Trauma

As mentioned, despite the decline in amputations due to trauma, trauma remains the second most common cause of amputation in the U.S. The mechanism of injury is predominantly blunt force, although penetrating injury can also lead to amputation and typically results in a more severe injury overall [3]. In an analysis of nearly one million records from the National Trauma Database spanning 2000–2004, Barmparas and colleagues found 8910 amputated patients and 151 incidents of multiple limb loss (7.3 %), most often the result of motor vehicle collisions (45.7 %) or railway accidents (19.9 %) [3]. Those with multiple limb loss were dominated by bilateral lower extremity amputation (96 out of the 151, or 63.6 %), followed by unilateral upper and lower extremity amputation (21.2 %), and bilateral upper limb amputation (11.3 %). Only six suffered amputation of three limbs (3.9 %). The average age of those injured was 37.2 years old, significantly younger than those who typically have dysvascular-related amputation. Trauma-related amputation demographics demonstrate two peaks in the age distribution for men (ages 20 to 29 and another from ages 70–79), and only one peak for women (70–79) [17]. Injuries in latter ages are probably most related to falls.

Although not the most pre-dominant cause of multiple amputations, the current military conflict in Iraq and Afghanistan, comprising Operations Iraqi Freedom (OIF), Enduring Freedom (OEF), and Operation New Dawn (OND), has perhaps brought traumatic amputation to the forefront of all etiologies. The most recent reports from these conflicts indicate that 1648 individuals have suffered major limb amputations (excluding digits), with 510 (nearly 31 %) of these individuals losing more than one limb [18]. Five wounded soldiers from these most recent conflicts underwent quadrilateral amputations, which necessitated upper extremity amputations proximal to the wrist. Although not counted in the quadrilateral totals, several service members who sustained amputations of three limbs also suffered severe injuries to the remaining extremity that resulted in severely debilitating partial hand amputations. The injuries encountered are typically secondary to blast injuries and are usually accompanied by a host of comorbidities ranging from additional fractures, soft tissue damage, and peripheral nerve injury to traumatic brain injury (TBI), post-traumatic stress disorder (PTSD), and other behavioral health problems [19]. Additionally, these patients are typically significantly younger than even those civilians who have suffered traumatic amputations. While only 18 % of service members with amputations during the Vietnam conflict sustained more than one amputation, it is believed that the increased rate has been the result of increased survivability due to improvement in immediate care, coordinated and rapid evacuation, and improved body armor, among other things [20]. Of particular concern, in their review of trauma patients, Barmparas and colleagues found that although loss of a single limb did not affect the rate of mortality from injuries, even after stepwise logistic regression, the requirement for multiple amputations was one of only four independent risk factors for mortality in amputated individuals [3]. The odds of mortality were increased by 164 % for those with multiple amputations; this was second to only Injury Severity Score in determining odds of mortality. It was a significant indicator of potential for death following amputation.

### Cancer

Limb loss as a result of cancer is rare in comparison to both vascular disease and trauma. When it does occur, it is most often the result of malignant bone tumors, which comprise 6 % of all cancers in those less than 20 years old [2, 21]. Osteosarcoma and Ewing sarcoma are the most common bone malignancies in the long bones and central axis, although other cancers have also been implicated in leading to amputation. Cancer-related amputations are most likely in the lower limb (76.1 %); above-knee and below-knee amputations alone comprise more than a third of all amputations caused by cancer [10••, 21]. Multiple amputations as a result of cancer are exceedingly rare. No difference in gender or race has been noted. The rate of amputations caused by malignancies has been decreasing along with traumatic amputation, most likely due to advancement in early detection of cancers and improvements in their treatment.

### Congenital

Congenital limb deficiency can be the result of genetic variation, exposure to environmental teratogens, or gene-environment interactions [2]. The effects can range from the loss of a finger or two to full bilateral limb loss. The rate of any amputation at all is very low, comprising about 0.8 % of all amputations, consistently, over a 10 year period, and perhaps 26 per 100,000 live births, and multiple limb loss is incredibly rare [10••]. Upper limb loss is slightly more common, making up about 58.5 % of known cases [10••].

## Medical and Surgical Management

The principle medical and surgical goals when caring for an individual with multiple limb loss include the simultaneous treatment of the underlying disease or trauma necessitating amputation, as well as optimizing the resultant residual limb to support independent function with or without a prosthesis. For patients who sustained multiple limb traumas, initial resuscitative interventions are focused on intervening to preserve life and limb. Immediate primary amputation and closure should be avoided in circumstances of “dirty” wounds, such as combat trauma, as immediate closure often results in wound dehiscence and infection. When managing a patient with severe extremity trauma and contaminated wounds, once the bleeding has been controlled, all efforts should be made to preserve as much viable tissue as possible and an appropriate dressing be placed over the limb. Preserving as much of the limb as possible in the early trauma resuscitation period will help support future reconstruction efforts, adequate soft tissue coverage of the residual limb at final closure, and even potential harvesting of donor tissue from one limb to preserve another. Serial dressing changes and soft-tissue irrigation and debridement are performed under anesthesia and are repeated over a period of days to weeks to longitudinally evaluate the viability of underlying tissues prior to performing definitive amputation and wound closure. The decision to close the wound is often based on the surgeon’s observation of the quality of the underlying tissue, including viable perfusion and absence of infection. Despite best efforts, however, infection rates of traumatic amputations may be as high as 23.2 % [22]. Other challenges from the protracted hospital stays for trauma victims and the frequent trips to the operating room include supporting adequate nutrition, ensuring appropriate pain control, and mitigating risks of secondary complications such as venous thrombosis, pulmonary embolism, joint contractures, pressure ulcers, disuse atrophy, osteopenia, and deconditioning. Therefore, an integrated trans-disciplinary comprehensive team approach to care is imperative to coordinate.

Considerable debate continues to exist surrounding the surgical indications for limb-salvage versus amputation in the setting of severe multiple extremity trauma. Limb salvage attempts are often complicated by prolonged hospital stays, multiple surgeries, and extended rehabilitation. In addition, despite best efforts at limb-salvage, amputation may still be necessary, especially if the salvaged limb continues to be a source of poor function, recurrent infections, and/or chronic pain. In contrast, early amputation and immediate prosthetic fitting may facilitate a more rapid hospital discharge, return to functional independence and improved quality of life [23••, 24•, 25]. In addition, current advances in prosthetic materials and components allow the accommodation of various residual limb lengths and shapes, facilitate improved socket comfort, and enhance rapid progression through rehabilitation.

Despite these strong supporting arguments for expeditious amputation, considerable caution and consideration must be observed when debating definitive amputation of any limb in the presence of existing limb loss. In the face of multiple extremity trauma where amputation has already been necessary in one limb, all effort should be made to save any possible other limbs because of the added challenges the patient will encounter when recovering from multiple limb loss. This is especially true for the individual with severe upper limb trauma; as a sensate hand, even with restricted motor function, may offer greater advantages than a prosthesis, in terms of performing activities of daily living or helping to don and doff a prosthesis of another limb. While there have been many advances in current production prosthetic technology, upper extremity prostheses still do not communicate pressure, pain, or hot or cold sensitivity, limiting external sensory feedback. This reduced sensory feedback is only compounded in patients suffering bilateral upper extremity limb loss. Similarly, salvage of one lower limb in the presence of amputation of the contralateral limb, may provide enhanced balance and stability to facilitate transfers, especially in situations where there might be tight spaces or when the individual’s prosthesis is broken or otherwise unavailable, such as in the middle of the night or when residual limb complications prohibit prosthetic use. Furthermore, just as there have been advances in prosthetic technologies, advanced orthotics now allow individuals with unstable, paralyzed, or even fused joints to resume even the most advanced activities [26]. Therefore, prior to performing definitive amputation, the surgical team should consult various surgical subspecialists to consider all possible options for limb reconstruction. They should also fully engage the rehabilitation team to discuss the implications of any reconstructive surgery, especially when considering transferring muscles from one area of the body to another, as this might ultimately compromise functional recovery, such as performing latissimus, gluteal flaps, etc.). In addition, a full discussion should take place between the surgical and rehabilitative teams with the patient regarding their functional expectations, vocational and avocational goals. This should also include a discussion of existing prosthetics and orthotic options. When possible, arranging visitation with other prosthetic or orthotic users can be very helpful.

Medical and surgical decisions for the patient with underlying vascular disease and/or advanced diabetes, especially for those requiring a second amputation, are also very complex and necessitate inter-disciplinary care between the surgical, medical, and rehabilitative teams. Because of the high likelihood of co-existent disease in other organ systems, consideration must be given to all factors influencing patient outcomes. Of primary concern is the presence of underlying cardiac disease, especially if ambulation is pursued after bilateral lower limb amputation, as the energy cost of ambulation increases significantly with bilateral lower limb amputation. For example, it has been estimated that the energy cost of ambulation for individuals with bilateral trans-femoral amputations increases nearly threefold [27] (Table 1). This greatly expands the burden experienced by the cardiac system during ambulation. Furthermore, in contrast to the traumatically acquired amputation, the likelihood of successful ambulation for an individual with bilateral transfemoral amputations resulting from vascular disease is extremely low [28]. Therefore, precaution must be taken when counseling patients and families that may have unrealistic goals. Consequently, successful rehabilitation requires an extensive cardiac evaluation and treatment regimen, including pharmacological and medical management to optimize cardiovascular function in order to allow participation in cardiac as well as ambulation rehabilitation. Precautions should also be established in terms of identifying target heart rate, blood pressure, and oxygenation levels to modify activities or supplement with oxygen as needed. Similarly, patient education and interventions should be employed to promote smoking cessation, appropriate dietary changes, and weight management. Finally, the medical team should exercise caution when using medications to manage issues such as pain, sleep and mood, as underlying hepatic or renal dysfunction may complicate their use.

As a general guiding principle for patients with both disease- and trauma-related amputation, every effort should be made to preserve as much residual limb length as possible, particularly in the presence of multiple limb loss. This is especially true when considering amputation above or below the elbow or knee. As discussed earlier, the energy demands for ambulation with a trans-femoral prosthesis are much greater than for the transtibial level, with estimates approaching nearly double the energy demand [29]. With regard to upper limb prosthetic function, abandonment rates of prosthetics increase greatly the more proximal the limb loss [30]. In general, a longer residual limb creates more mechanical advantage as well as preserves more of the remaining musculature to generate power. In addition, the resultant larger residual limb surface area allows for greater prosthetic socket interface, dispersion of forces, and overall socket comfort. The possible exceptions to the principle of preserving as much residual limb length as possible include the decision to perform a knee disarticulation versus a high transtibial amputation in non-ambulatory patient (because of underlying paralysis, dementia or other diseases) or a long transtibial amputation instead of a Symes amputation in a very active patient, who has the goal of returning to high level sports/recreation. For the non-ambulatory patient, amputation at the high transtibial level may result in flexion contracture of the knee, leading to impaired transfers and secondary skin breakdown with subsequent pressure and friction. For the very active patient, amputation at the long transtibial length versus the Syme’s level will provide more prosthetic options with dynamic response, multi-axial feet, or the addition of shock absorbers or torsion control components that can accommodate a wide variety of activities [31, 32]. A similar argument could be considered for patients that may benefit from a powered ankle/foot prosthesis, which is currently unavailable at the Symes amputation, but has the potential to dramatically enhance biomimetic lower limb function after transtibial amputation independent of underlying etiology [33, 34], although further evidence is needed to demonstrate its efficacy across multiple patient populations.

Finally, when optimizing the residual limb for any amputation, including those with multiple limb loss, careful attention must be made to achieve adequate soft tissue coverage, optimal balancing of muscle forces through myodesis and myoplasty, appropriate beveling of distal bony prominences, and management of distal nerves to help mitigate the effects of neuroma formation. Achieving optimal muscle balancing around residual limbs is critically important for patients with multiple limb loss to avoid joint contractures and misalignments which will likely disturb proper prosthetic fit and function. In addition, for patients with multiple limb loss that will return to higher level activities or be at a higher risk for falling, adequate anchoring of the myodesis is very important to help prevent future failure. Achieving soft tissue coverage can be extremely challenging when performing an amputation within the zone of injury for trauma patients or for those with compromised soft tissue because of vascular disease. Skin traction devices or vacuum assisted dressings may be helpful [35, 36].

When managing distal bones, care must be taken to shorten the fibula in relationship to the distal tibia to facilitate a more conically shaped residual limb. For individuals with bilateral upper limb loss at the trans-humeral level, a distal angulation osteotomy of the humerus as described by Marquardt should be considered, as it will enhance more intimate prosthetic suspension and control, and facilitate independent volitional control of internal and external rotation [37•]. In addition, for patients with bilateral upper limb loss at the transradial level, especially those with vision loss or impairment, a Krukenberg amputation should be considered [38]. Finally, as novel interface strategies and advanced prosthetics become available, prior to cutting peripheral nerves, surgical teams should consider the possibilities of performing contemporaneous or reconstructive surgeries such as targeted muscle reinnervation to enhance prosthetic control or manage neuromas, as well as implanting electrodes to provide more reliable and intuitive prosthetic control interfaces [39, 40].

## Pain Management

Early and aggressive pain management, including early mobilization following significant trauma or major surgery, is thought to shorten rehabilitation lengths of stay and reduce the risk of developing chronic pain [41••]. For patients with multiple limb amputation, a multi-modal approach to pain management is achieved by integrating a variety of interventional procedures, oral and intravenous medications, physical modalities, and complementary and alternative medicine approaches.

### Pharmacotherapy

While opioids are effective for the treatment of acute pain following trauma and multiple surgeries, their use should be monitored closely as their side-effects respiratory depression, constipation, nausea, ileus, immunosuppression, tolerance, dependence, as well as opioid-induced hyperalgesia [42]. Often the overall opioid use can be minimized by combining other pharmacological agents, which target various sources of nociception, such as regional anesthetic agents, anti-epileptics, non-steroidal anti-inflammatory drugs, and anti-depressants. The effectiveness of this approach had been demonstrated by multiple studies, with a meta-analysis noting a 15–50 % decrease in morphine PCA (patient-controlled analgesia) dosing among studies evaluated [43]. For severe pain, including multi-trauma combat casualties, particularly combat casualties, oral transmucosal fentanyl citrate (OTCF), and/or Ketamine have been used in the acute setting, however, Ketamine is recommended for individuals with multiple limb amputation as it poses lower risk for worsening hemodynamic instability [44].

Non-steroidal anti-inflammatory drugs (NSAID’s) and the related class of cyclooxygenase-2 (COX-2) inhibitors can be effective for mild to moderate pain or utilized with the above strategy in the setting of severe pain. In a patient with limb loss, these medications may also reduce the rate of heterotopic ossification formation at the residual limb. NSAID use, however, for patients with multiple fractures, particularly spine fractures, should be used cautiously because of their potential negative effects on bone healing [45]. In addition, their use may be contraindicated for patients with renal impairment or high risk for gastrointestinal bleeding.

Ketamine has become a common anesthetic agent in pain management of patients with limb loss, especially in the military. Ketamine is often used not only far forward on the battlefield and peri-operatively to reduce the risk for a chronic pain state, but also post-operatively at lower doses as part of multimodal analgesia. The current mechanistic theory includes NMDA receptor antagonism which can inhibit the development of central sensitization and chronic pain [46]. Other agents given with NMDA receptor antagonistic properties exist, although it is unclear how effective these agents are at reducing chronic pain. A recent systematic review of post-surgical pain following amputation failed to demonstrate a reduced incidence of chronic pain with the use of Ketamine [47]. Side-effects even at low doses may include nystagmus and hallucinations.

Gabapentin is a neuroleptic medication that has been used safely in patients with multiple limb loss for neuropathic pain both at the amputation site as well as for phantom limb pain for some time and is considered by many to be a first-line treatment for these conditions. Although its role in neuropathic pain has not been fully elucidated, it is known that gabapentin has no direct GABAergic action, neither directly blocking GABA uptake nor metabolism. The greatest amount of evidence points to gabapentin antagonism of NMDA receptors, antagonism of calcium ion channels of the central nervous system, and inhibition of peripheral nerves as the most likely way by which it can help to attenuate neuropathic pain [48]. In application, it should be started at a low dose and titrated while monitoring for orthostatic hypotension. Its effects are often not apparent until doses over 600 mg three times daily and the dose should be titrated further prior to changing agents. For patients who only report a partial benefit from Gabapentin, pregabalin may be effective because of its higher affinity for the same receptors [49]. Tricyclic antidepressants (TCAs) are another class of medications used for neuropathic and phantom limb pain, with the additional benefit of somnolence in the setting of a patient with nocturnal pain and/or insomnia. These medications are believed to relieve neuropathic pain primarily through inhibition of presynaptic reuptake of serotonin and noradrenaline [50]. Similarly, medications such as selective serotonin and norepinephrine reuptake inhibitors (SNRIs), like duloxetine, have multiple indications for use, including depression, neuropathic pain, and most recently chronic musculoskeletal pain. The combination of the above classes of agents, along with regional anesthesia such as epidurals and peripheral nerve pain catheters, may reduce not only acute but also chronic pain in those with traumatic as well as vascular amputations [47].

### Non-pharmacological Intervention

Often complex pain syndromes, such as phantom, residual limb, and neuropathic pain can persist despite aggressive pharmacological interventions; however, the simultaneous application of physical modalities, adjuvant therapies, psychosocial support, and early mobilization can have an additive effectiveness. Most modalities use physical energy for their therapeutic effect, including heat, cryotherapy (cold), TENS (transcutaneous electrical nerve stimulation), and acupuncture. While TENS and acupuncture are not universally accepted as systems for the management of neuropathic and phantom pain, numerous reports have demonstrated the effectiveness of acupuncture in these areas [51–53] while a recent study showed a mean reduction in pain intensity scores of 1.8 at rest and 3.9 on movement in five patients after just 60 min of TENS [54]. Each patient in the latter study had either phantom pain, residual limb neuropathic pain, or both. In addition, individuals with limb loss are taught desensitization techniques, such as rubbing or tapping methods, which can be performed with an assistive reaching device as needed.

Mirror therapy is another non-invasive therapy that has been evidenced to reduce phantom limb pain. This treatment requires the patient attempt movements of the amputated limb while visualizing the same movements of the intact limb via a mirror placed over the amputation site. One study reported 100 % of their subjects (22 patients) experienced pain reduction of -24 mm on the VAS after 4 weeks of daily 15 min treatments [55]. It has been postulated that mirror therapy works by helping to reorganize the somatosensory cortex, as has been demonstrated on functional MRI [56]. Although individuals with bilateral limb loss may not be candidates for mirror therapy, the use of a virtual limb avatar may have similar benefits [57].

Early mobility through the use of an immediate post-operative prosthetic (IPOP) device has been reported to reduce phantom limb pain, although recent experience is limited [58]. This entails a plaster cast applied immediately post-operatively, that may also serve to prevent knee flexion contractures in the case of a transtibial amputation. Unfortunately, due to accessibility as well as the nature of the amputation event, use of an IPOP is often restricted for patients with complex trauma wounds, as seen in most combat casualties. Additionally, they are not a widespread component of standard of care currently, however, these isolated results indicate that they could be worthy of consideration and further evaluation. Even without an IPOP, however, early mobilization is still the goal and has several proposed mechanisms of action in reducing pain, including enhancing endogenous opioid release [59]. Furthermore the effects of immobility, such as kinesiophobia and fear-avoidance behaviors may contribute to the development of chronic pain, which is characterized more by patient distress [60].

## Behavioral Health Considerations

Behavioral health complication is a well-known phenomenon in persons who undergo limb amputation, especially those with multiple limb loss. Amputation imposes significant changes across multiple domains including function, comfort, and body image, which may have significant negative longstanding emotional consequences. The emotional intensity of limb loss has been compared to that of grieving death, as it represents the loss of not only bodily integrity but a sense of self [61••]. Therefore, healthcare providers must be sensitized to recognize the early signs and symptoms of behavioral issues in order to institute immediate treatment.

### Depression

Depression and depressive symptoms have long been recognized for their connection to increased activity restriction, poor self-rated health, and increased all-cause mortality rates [62]. While the literature on the prevalence of clinical depression or depressive symptomology in the amputee population is limited, and direct comparison between studies is difficult due to the lack of a common standard for measurement [63•], the presence of depression in this population is a well-recognized behavioral health issue. Recent studies have generally arrived at similar prevalence figures. In a national survey collecting data from 1998 to 2000, Darnall and colleagues found a prevalence of depressive symptoms of 28.7 % in a sample of 914 people taken from the Amputee Coalition of America, [63•]. Another study, involving 42 adults with amputation who underwent rehabilitation in a Portugal rehabilitation center, reported 31 % of subjects fulfilling criteria for the diagnosis of depression [64]. In comparison, the estimated prevalence of depression in the general population is 3.6 to 10.6 %, and 11 % for populations with chronic illnesses [65–67].

The incidence of depressive symptoms appears to vary with time. In their evaluation of 105 patients consecutively admitted into their inpatient rehabilitation service, Singh et al. found that the incidence of depression and anxiety dropped from 26.7 to 24.8 % respectively on admission to 3.8 and 4.8 % on discharge [68]. However, on a 2–3 year post-discharge follow-up survey, the incidence of depression and anxiety increased from 23.5 % for both on admission and 2.9 % upon discharge, to 17.6 and 19.1 % of each, respectively [69]. This finding suggests that while initial rehabilitation can provide patients with adaptive techniques, their positive effects may not be long-lasting, especially as patients return to community living. Regarding long-term outcomes, a review by Horgan and MacLachlin concluded that the depression prevalence increases between 1 and 2 years post-amputation, but decreases to prevalence comparable to that of general public by 10–30 years [62].

### Body Image Anxiety

Following amputation, body image and self-identity can be significantly challenged. Body image may be defined as “the combination of an individual’s psychological experiences, feelings and attitudes that relate to the form, function, appearance and desirability of one’s own body” [70]. Limb loss, especially multiple limb amputation, can significantly alter how individuals perceive themselves through their appearances as well as interactions with the external environment. While prosthetics may help an individual to adapt to the functional deficit, they may not make up for the change in body image. Poor body image has been reported to highly correlate with increased anxiety, lower physician satisfaction, and lower prosthesis satisfaction [71], while a higher level of body image anxiety correlates with a lower quality of life [72].

### Behavioral Health Issues Asssociated with Upper Limb Amputation

When compared to those with lower limb loss, individuals with upper limb amputation generally experience greater challenges with functional independence and cosmesis. Furthermore, individuals who sustain an upper limb amputation are more likely to be younger than those who sustain lower limb amputation, which itself presents additional challenges with body image, initiating relationships, and vocational rehabilitation [73]. Despite the increased challenges faced by the upper limb amputees, the limited literature suggests that the prevalence of depression for individuals with upper limb amputation is comparable to that associated with lower limb loss. The analysis of data from the UK military service members with upper limb amputations shows depression incidence at 28.3 % [73], a number that is comparable to the 32 % reported in the general United States for upper limb amputees and 28.3 % for lower limb amputees [63•]. A recent study, however, from Desteli et al., contradicted this finding, reporting that individuals with upper limb loss had increased difficulties in social adjustment, adjustment to functional limitations, as well as anxiety, depression, and body image disturbance, which was worsened when the dominant hand was involved [74].

### Behavioral Health Issues in Combat Amputees

Combat casualties with limb loss represents a unique patient population. Compared to civilians, combat amputees (1) are generally younger and have higher baseline fitness, (2) received their amputation due to high-energy blast injuries as opposed to peripheral vascular disease in their civilian counterpart, (3) frequently have constellation of other physical and psychological sequalae such as polytrauma, TBI, depression, and PTSD, and (4) nearly all undergo a long period of care at specialty rehabilitation centers. Given these differences, the discussion of the behavioral issues of combat amputees requires separate consideration. A retrospective review of 382 amputees from the military database and 274 non-amputee combat injury patients between 2001 and 2005 showed that, although the two groups had similar injury severity scores (ISS), the amputee group had significantly increased rates of any mental health diagnosis compared to non-amputees, including non-organic sleep issues, pain disorders, and post-concussion syndrome [75]. Long-term depression and PTSD prevalence of combat amputees from OIF and OEF have been reported to be 24.0 and 58.7 % respectively [76]. The length of time from injury to amputation appears to be an important factor in psychological health outcome. A retrospective analysis from 2001 to 2008 revealed that combat casualties who underwent delayed amputation (more than 90 days after injury) had significantly higher rates of PTSD, adjustment disorders, anxiety, mood disturbances, and substance abuse [77].

### Rehabilitation Considerations to Limit Behavioral Health Issues

Rehabilitation strategies to help mitigate the negative behavioral health effects of amputation should seek to maximize the protective factors while minimizing antagonizing factors. It has been shown that a sense of hope, higher education level, higher rank (for service members), family and social support structure, and better problem-solving skills are associated with improved behavioral health outcomes [22, 23••, 73]. Conversely, avoidant coping strategy, poor family and social support structure, living at near-poverty level, having comorbid conditions, poor pain control, and phantom limb pain are associated with poor behavioral health outcomes [23••, 73]. Specialized rehabilitation centers, such as that created by the U.S. military, serve as excellent models for the integration of holistic care for individuals with limb amputation(s) [78]. Having centralized treatment facilities promotes a sense of community and helps foster camaraderie amongst similar patient groups. Peer visitation and support groups can also help maximize the social support structure [79]. A well-organized interdisciplinary treatment team allows for open discussion between providers and patients and promotes the prompt recognition and treatment of behavioral health issues to minimize their lasting effect.

## Rehabilitation Strategies for Individuals with Multiple Limb Amputation

Rehabilitative interventions for patients with multiple limb amputation should be initiated during the acute hospitalization and when possible, even before definitive amputation. As in many other medical disciplines, optimal comprehensive care is achieved through well-coordinated interdisciplinary interventions by a team of experienced specialists. Given the unique challenges posed by patients with multiple limb loss, teamwork is fundamental to achieving rehabilitative success in these individuals. Critical members of the interdisciplinary team include the surgeon, physiatrist, physical therapist, occupational therapist, prosthetist, nurse, case manager, social worker, behavioral health specialist, dietitian, and assistive technology specialist. Depending on the individual needs of each patient, other specialists may include oncology, cardiology, speech language pathology, recreational therapy, neuropsychology, etc.

The complexity and number of problems frequently associated with multiple limb loss, both from trauma and disease, often leads to confusion for the patient and family, especially with the number of specialists caring for the individual. It is apropos, therefore, that a single provider be designated as the coordinator of information and communication with the patient and conduct regular inter-disciplinary team meetings as well as family meetings to ensure that all members of the team, including the family, are all working on a common set of goals and are fully familiar with the ongoing medical, surgical, behavioral health, and rehabilitative issues of each individual patient. It is not infrequent that one member of the team might observe a particular behavior or comment by a patient during a therapy session that would be informative and helpful to the treatment being carried out by other members of the team. For example, the use and dose of under or over-the-counter medication for conditions such as pain may have a significant impact on the patient’s ability to participate in rehabilitation sessions. Additionally, as is frequently observed in multi-trauma, other associated injuries that were missed during the initial trauma screen may become apparent during rehabilitation. In particular, remote musculoskeletal injuries such as fractures or ligament sprains typically become readily apparent during initial weight bearing or engaging shoulder activities such as overhead reaching for dressing or bathing. Similarly, the residual effects from an undiagnosed TBI may manifest as trouble with memory, concentration, executive functioning, disturbed balance, headaches, or even behavioral changes such as irritability and/or mood liability [80]. These conditions often warrant further evaluation and dedicated treatment and can impact treatment in all areas of the rehabilitation.

Early rehabilitation efforts should involve substantial patient education, including helping to shape the patient and family’s expectations, particularly regarding prosthetic devices and subsequent training to avoid unrealistic expectations. In addition, significant emphasis must be placed on the importance of other general health concerns to include smoking cessation (as indicated), proper skin care (especially around the affected areas to avoid skin breakdown), cardiovascular conditioning, and optimizing strength and range of motion. Contracture formation for immobilized patients can occur quickly and can have a significant negative impact on prosthetic use and function. Strategies to prevent contractures include range of motion exercises, lying prone to prevent hip flexion contractures, sand bag placement under the residual limb for patients with transtibial amputations to prevent knee flexion contracture and by the side of the thigh in transfemoral amputees to prevent hip abduction contracture. All members of the treatment team as well as the patient’s family should take an active role in enforcing these strategies. Peer visitation by other individuals with similar patterns of limb loss can be extremely helpful not only during the acute rehabilitation phase, but through the entire continuity of care [81, 82••].

Prosthetists should visit during the acute inpatient phase to discuss the various prosthetic options available. At this time, they should also perform initial assessments for preparatory prostheses or assistive devices and ensure the proper fitting of residual limb compression socks (“stump-shrinkers”) or silicone sleeves. These latter components, compression socks or silicone sleeves, should be applied as soon as possible after definitive amputation and wound closure to help with residual limb edema control, residual limb maturation, and protection, all of which are important in the transition to eventual prosthetic socket fitting. Typically, once the patient is cleared by the surgical and medical staff, they will be fit with a thermolyn plastic socket within 24 hours. That same day they will begin training with their initial prosthetic device(s). Initially, the wear schedule for these components will only encompass the hour or so spent training in physical therapy and begin to increase with patient comfort beginning a few days thereafter. In some practices, the application of a rigid dressing in the operating room after amputation can be applied, especially if edema control and protection are of increased concern. The addition of a pylon and prosthetic foot to the rigid dressing, in the form of an Immediate Post-Op Prosthesis (IPOP), has been used to facilitate early standing; however, this should be instituted with caution depending on the surgeon’s comfort with the soft-tissue integrity and viability of the residual limb [83]. In summary, the general rehabilitation principles that should be applied to all individuals with multiple limb loss include: (1) comprehensive evaluation of motor, sensory, cardiovascular, and cognitive function; (2) extensive patient and family education; (3) evaluation of skin integrity, especially of all residual limbs and support of optimizing wound healing through dressing changes, edema control, stump maturation and protection, as well as skin/scar mobilization; (4) full assessment of pain conditions, including pharmacological and non-pharmacological management strategies to help facilitate full participation in rehabilitation; (5) functional assessment of activities of daily living (ADL’s) and mobility; (6) patient engagement to establish meaningful short and long-term goals, with breakdown of the sub-tasks of each goal to ensure success; (7) initial prosthetic component selection; (8) specific training on donning/doffing the prosthesis as well as maximizing its’ use; (9) gradual advancement from basic activities such as transferring, standing and walking to more advanced activities such as negotiating stairs, curbs, and obstacles; (10) specialized adaptive equipment or assistive technology to promote independent living, driving, and return to vocation/sports and recreational activities, including proper wheelchair selection, fitting and training when indicated; and (11) active encouragement and assistance with promoting full participation in society and the highest quality of life. Inherent in each of these principles, is the fundamental importance of developing a strong alliance with the patient and their family that is fostered through active listening, encouragement, advocacy, compassion, and professional expertise. The following sections outline some of the unique considerations with caring for sub-populations of patients with multiple limb loss.

### Bilateral Upper Limb Loss

Amputation of the upper limb most commonly occurs as the result of trauma, including industrial accidents, and generally represents a younger patient population than individuals with lower limb amputation (more often resulting from disease manifestation). As mentioned previously, the psychological implications of limb loss are substantial and may be significantly magnified when the limb loss occurs at a young age or effects one or both upper limbs. Unlike inpatients with unilateral upper limb amputation where the intact limb becomes the new dominant hand and the prosthetic side is primarily used for assistance, individuals with bilateral upper limb loss become dependent on at least one of their prosthetic devices to become their dominant method of upper limb manipulation. Typically, this is the longer of the two residual limbs, particularly for patients with both above and below elbow amputations.

Early rehabilitation efforts are focused on maximizing the patient’s functional independence, typically progressing from *self*-*care* (personal hygiene, eating, grooming, dressing, and toileting) to *communication skills* (writing, phone and computer use), *homemaking* (cooking, cleaning, laundry, child care), and *social skills and avocational interests* (sports, recreation, education, driving, etc.) [84]. Well skilled Occupational Therapists (OT) become critical treatment team members, often conducting multiple therapy sessions per day at the bedside and in therapy clinic to help maximize patient independence both with and without prostheses. A close relationship and frequent communication is required between the occupational therapist (OT) and the prosthetist, as frequent modifications to the prosthesis may be required to facilitate optimal operation and training. The relatively low incidence and prevalence of upper limb loss as compared to lower limb loss in the United States means that there is generally a paucity of prosthetists with extensive experience in upper limb prosthetic fabrication and fitting. Even though there have been considerable advancements in externally powered (myoelectric) prosthetics, most individuals with bilateral upper limb loss prefer the use of a body-powered prosthesis, because of their lighter weight, reliability, and biofeedback through the Bowden cable system. In addition, most patients prefer the functional versatility of a hook rather than a hand terminal device, although an electric hook, such as the Griefer [85], may be substituted on one side when the patient returns to an occupation or activity that requires greater grip strength. For patients with shoulder disarticulation or very short transhumeral amputations, an externally powered myoelectric or switch controlled electrically powered elbow and/or terminal device is likely needed in order to reduce the effort required for prosthetic operation and prevent extensive fatigue.

Fundamental to successful prosthetic use is the ease of successful independent donning and doffing. This is challenging for the individual with bilateral upper limb amputation and typically requires the integration of bilateral harness systems. For the transradial amputee, utilizing a control attachment strap or cord that connects to the front support strap of the contralateral prosthesis and from there is either sewn together or run to a center ring, can typically facilitate this need [84]. A similar principle can be employed for patients with transhumeral amputations, wherein the control suspension strap of one prosthesis is used as the front suspension strap of the contralateral prosthesis, allowing independent prosthetic function. The lateral suspension straps and elbow/terminal device control mechanisms can be attached to the harness in the conventional manner. Donning and doffing of the harness can typically be achieved in a similar fashion to putting on a coat, and the patient is taught to remove the prosthesis to a place/position ready for re-donning, such as an appropriate height wall mounted hook or rack.

Another common approach for bilateral upper extremity prosthetic users with one transradial and one transhumeral amputation is to use a myoelectric prosthesis on their transradial side and body-powered prosthesis on their transhumeral side. The use of a myoelectric prosthesis on the transradial side allows for independent donning and doffing as well as unrestricted shoulder and elbow mobility and increased grip strength. Use of the body-powered prosthesis on the transhumeral side provides increased fine motor dexterity and feedback through the cable system. This option is chosen most by bilateral upper extremity patients with bilateral lower extremity amputations. Ultimately it can be difficult to predict which type of prosthesis patients will incorporate into their self-image, ADLs, and avocational and vocational pursuits. Many patients thought to be great body-powered upper extremity prosthetic user often prefer myoelectric systems and vice versa. Patients often use one type of prosthesis for one activity and another prosthesis for other activities (i.e., body-powered prosthesis for gardening or shoveling snow, a myoelectric prosthesis for work or business situations, and an activity-specific prostheses for avocational activities). No single prosthetic option fits all the user’s needs to have complete rehabilitative outcomes that approach normalization as compared to other individuals in their age and gender categories. Therefore, it is important to present and allow patients the option to use prostheses from several categories of upper extremity prostheses. Upper extremity prosthetic devices generally fall into the following categories: (1) passive, (2) body-powered, (3) hybrid, (4) myoelectric, or (5) activity-specific prostheses. No group is completely exclusive unto itself, sometimes specialty rehabilitative upper extremity prostheses need to be fabricated. For example, when upper extremity patients with bilateral lower extremity transfemoral involvement learn to ambulate, custom upper extremity crutch prostheses may need to be fabricated. If the patient is learning to walk with bilateral transfemoral prostheses and the prosthetic knee(s) collapses the patient will need to support themselves with a prosthetic device. Most, if not all, prosthetic elbows have a manufacturer recommended weight limit of 50 lbs. If the prosthetic knee collapses and the user exceeds the weight limit of the prosthetic elbow, serious injury could result to the patient, and ultimately delay the rehabilitation process. For these individuals, specialty crutch prostheses need to be fabricated without the use of elbow joints so that the patient can safely support their body weight during the initial ambulation process in case of knee failure. Once the patient gains stability on their lower extremity prosthetic devices, the use of crutch prostheses can be discontinued and normal daily use prostheses can be incorporated into ambulation.

In order to achieve successful prosthetic control for bilateral upper limb amputees, early rehabilitation efforts should be focused on the evaluation of proximal joint range of motion and strength. Inasmuch, the OT needs to be fully aware of the joint motions necessary to control a body-powered prosthesis. Targeted range of motion and strengthening exercises can begin even before the residual limb is ready for prosthetic fitting. Full range of motion and strength of the shoulder girdle and upper trunk are critical for successful control of the harness and cables in order to perform voluntary elbow and terminal device movements. In general, the same motions that control elbow flexion are utilized to control voluntary hand opening (i.e., scapular abduction, shoulder flexion). Transfer of control between the elbow and terminal device is achieved through an anterior locking mechanism that is generally activated by scapular adduction and/or shoulder extension. For patients who use an externally powered prosthesis, particularly those with higher level amputations, myoelectric control sites should be identified early (typically at the deltoid, biceps, triceps, wrist flexor/extensors, depending on the amputation level). Initial training of myoelectric control strategies can be initiated using computerized biofeedback devices before prosthetic fitting. As mentioned previously, however, for patients with bilateral upper limb loss, initial prosthetic fitting is often begun with training with at least one body-powered prosthesis and transitions into proficient use of myoelectric control. As discussed, it is important to give the user the choice of the prosthetic device that best suits their needs for independence and normalization as it is often difficult to predict a patient’s preferred prosthetic usage for any given ADL, vocational, or avocational tasks. As upper limb prosthetic technology continues to advance with the recent development in the fields of implantable electrodes and direct neurological control, the approach to and the timeline of prosthetic fitting may evolve. Everyone involved in the amputee’s care should make every effort to be apprised of all advances in technology and the prosthetic science to ensure the highest quality care for their patients in line with the best available tools.

Performing midline activities to support self-care is essential to the process of rehabilitation. Therefore, special attention must be applied to the individual with bilateral upper limb amputation to promote a maximal range of motion and the incorporation of wrist-flexion components into the prostheses. This is typically achieved through the use of the Five Function body-powered wrist that incorporates supination-pronation, wrist flexion–extension, and a quick disconnect wrist to change terminal devices, which can be actively positioned with the Bowden cable system or passively positioned by the user by applying the necessary forces from the contralateral limb, wall, table or other fixed object. Similarly, developing the greatest degree of elbow flexion possible is also important in enabling the manipulation of objects and to support grooming and eating. Myoelectric prostheses for bilateral upper extremity patients incorporate the use of electric wrist rotators, passive wrist flexion units, and quick disconnect wrists to facilitate changing to other terminal devices, to allow patients to get to midline for ADLs. Self-suspending prosthetic sockets are the most common way to fit both body-powered and myoelectric transradial prostheses. Gaining suspension of the prosthesis from the patient’s boney anatomy around the elbow can be a very effective way to suspend the prosthesis. To increase stability for high-demand activities, a single pivot elbow joint can also be used for individuals with longer transradial amputations, whereas a polycentric hinge is more appropriate for the individual with a very short residual limb or otherwise limited elbow flexion. Step-up hinges, transradial-activated locking hinges, and outside locking hinges can also be employed for those with very short residual limbs or a limited range of motion. Although a polycentric elbow hinge may be less stable and require more maintenance, the enhanced elbow range of motion may make a significant difference in terms of supporting functional independence. Furthermore, the alignment and length of the upper limb prosthetic devices can be adjusted to better facilitate midline activities. For patients with a transradial amputation, the socket and terminal device may be set in up to 30 degrees of forward-anterior alignment and set medially as is functionally desired and cosmetically acceptable. Transradial activity-specific prostheses will often incorporate the use of locking silicone liners with either a pin lock or lanyard suspension. In comparison, for patients with above elbow amputations, forearm length can be shortened to reduce the overall weight of the prosthesis, which decreases the amount of force needed to flex the elbow, and brings the center of gravity closer to the body to enhance control. Suspension for transhumeral prostheses is usually achieved through the use of an intimately fit suction socket, although locking silicone liners with a pin lock or lanyard can also be employed. For patients with shoulder disarticulations or high transhumeral neck amputations, the prosthetic shoulder joint should be slightly internal. Locking shoulders can assist patients with the ability to lock the shoulder in flexion for activities at different heights and overhead. Internal and external humeral rotation can be accomplished with an elbow turntable or lamination collar. Most of the prosthetic shoulders in production also allow abduction and adduction through a friction pivot built into the shoulder joint. Just as it is difficult to predict which prostheses an individual will choose for ADLs, it can also be difficult to predict the terminal devices an individual will select to for ADLs. Most prostheses, body powered along with myoelectric, are fabricated with quick disconnect wrists to facilitate the changing of terminal devices for different activities. Some of the more common body powered terminal devices include the Hosmer 555, 5Ti, VO hand, V2P, and Grip III. Some of the more common myoelectric terminal devices include the Otto Bock VariPlus, Otto Bock Greifer, Motion Control ETD, iLimb, and the BeBionic. In general, it is best to think of upper extremity prosthetic devices and terminal devices as tools in a tool box, patients will use the most appropriate tool for the task or activity at hand.

Successful control of upper limb prostheses requires frequent practice and patience on the part of both patients and rehabilitation teams. Successful therapy sessions are achieved through breaking down individual control tasks. This can be facilitated by engaging in preparatory training with a training prosthesis prior to definitive prosthetic fitting. Once the basic individual motion control skills have been achieved, patients are ready for the integration of these movements to perform functional tasks. This process often needs to be accelerated for the individual with bilateral upper limb loss because of the functional and psychological implications of being able to resume basic self-care activities as soon as possible.

### Bilateral Lower Limb Loss

As mentioned, ambulation following bilateral limb loss requires a substantial increase in energy input on the part of the user. As a result, resuming independent community ambulation can be very challenging, especially for individuals with amputation secondary to underlying peripheral vascular disease. The challenge only escalates with more proximal amputations. In light of this, although each patient may merit a trial with prosthetic training, realistic expectations have to be discussed with the patient with advanced cardiovascular disease and bilateral above-knee amputation. The energy expenditure necessary for these individuals to ambulate following amputation may in fact be greater than their health will allow. For the motivated and otherwise healthy patient with bilateral lower limb loss, community ambulation, running and full participation in sports and recreation are certainly possible and have been greatly facilitated by the recent advances in lower limb prosthetic devices. Under guidance, integration into sports and group activities can often aid the rehabilitation process by providing both physical benefits along with helping in the acceleration of psycho-social normalization.

Similar to the approach with upper limb loss, early rehabilitation for individuals with bilateral lower limb amputation begins with patient and family education. It is important for the rehabilitation team to outline the phases of rehabilitation, so the patient has realistic expectations of both the process of rehabilitation and the outcomes possible. Proper education regarding skin care, preventing joint contractures, mitigating the effects of deconditioning, and pursing a healthy life-style are critically important to enhancing outcomes and well-being. In addition, because of the increased risks associated with aging with limb loss, including a higher incidence of cardiovascular disease, diabetes, hypertension, arthritis, back pain, and over-use injuries [86•, 87], it is important to educate the patient on these risks and to implement measures to mitigate any modifiable risk factors. The phases of rehabilitation for individuals with bilateral lower limb loss typically progresses from pre-prosthetic conditioning, to prosthetic fitting and training, and then to gradual mobility training, advancing from standing to level ground walking, ramps and stairs, obstacle negotiation, and, finally, sports and recreational activities. Pre-prosthetic assessment and training should focus on a comprehensive evaluation of the patient’s musculoskeletal system, cognitive and psychological conditions, presence and quality of pain syndromes, and condition of the residual limbs. Proper skin and wound care should be initiated immediately, with active participation from the patient and family members in order to enhance education and promote independence, especially after discharge from the hospital. Compression “shrinker” socks or prosthetic liners should be utilized as soon as possible to promote donning and doffing skills, as well as to help control residual limb edema, protect the skin and soft tissues, and promote limb maturation and shape. Range of motion and strengthening exercises should be initiated at the bedside to promote core muscle maintenance and/or development and proximal muscle strengthening, as well as reducing and preventing any contractures of the hip and knees. If significant joint contractures develop, they will create difficulty with socket fitting and prosthetic alignment. In addition, joint contractures may lead to functional limb length discrepancies and biomechanical deficits, resulting in an asymmetric and inefficient gait which in turn can lead to a variety of negative secondary health issues [88]. Therefore, proper positioning of the lower limbs at rest should also be observed when the patient is in a bed or in a chair to prevent contractures. Devices, such as an extension board to be used when sitting in a chair or wheelchair may be needed to facilitate full knee extension.

Most lower-limb prosthetic devices are “passive,” meaning that all the power needed for mobility must be generated from the remaining musculature; therefore core strengthening of the trunk, abdominals, lumbar, and gluteal muscles is very important to promote successful prosthetic use. Similarly these muscles are also very important for proper trunk control and positioning in a wheelchair. As the upper limbs must be used for successful transfers (e.g., from a bed, commode, or wheelchair), special attention must also be focused on strengthening the patient’s triceps, pectoral, and latissimus muscles, as well as with teaching proper transfer techniques to preserve upper limb function and avoid injuries to the shoulder, elbow, or wrist, because the risk of overuse and entrapment nerve injuries are greater for amputee and wheelchair user populations [89]. When necessary, special equipment such as a sliding board may be indicated.

In prosthetic socket design for individuals with bilateral transfemoral amputation, it is important to avoid midline contact with the contralateral prosthesis and irritation of the groin and genitalia. A sub-ischial socket design should be utilized whenever possible and a suction or pin-lock suspension may be employed to avoid waist belts or auxiliary suspension systems, which can be cumbersome and contribute to prosthetic rejection. Special liners, such as the Sealin^®^ suspension liner may facilitate suspension and can be customized depending on the size and shape of the residual limb. For patients with very short transfemoral amputations or hip disarticulations, more creative sockets must be fabricated. These in particular will often require multiple adjustments by a well-skilled prosthetist working closely with the patient as they progress through rehabilitation and as their residual limb(s) change in size, shape, and tolerance. Early prosthetic training for individuals with bilateral transfemoral amputations is usually initiated with short bilateral prostheses. These typically include a check socket, short pylon, and flat, wide-bottomed foot. The lower height lowers the patient’s center of gravity, making it easier to stand and walk. Further, injuries as the result of falls and falls recovery, common in the initial phases of re-ambulation, are much easier for the patient. As the patient demonstrates improved stability and ambulation, prosthetic knee components can be added along with height. Combat casualties with bilateral transfemoral amputation have demonstrated positive outcomes with the use of bilateral microprocessor knees, which can be programmed throughout the phases of rehabilitation to adjust to the patient’s individual progress. The role of powered prostheses for individuals with bilateral lower limb loss has not yet been determined, however, the concept offers great hope for many individuals with mobility challenges, particularly as individuals age with their disability.

### Mixed Upper and Lower Limb Loss

In general, the same principles as mentioned previously apply to patients with mixed upper and lower limb loss. Additional considerations, however, include the unique challenges of donning and doffing one’s lower limb prosthetic device(s) with an absent hand or hands. In these cases, considerable problem solving is often required between the patient’s prosthetist and therapists to accommodate this with special devices or adaptive equipment. Axillary pull-tabs may be attached to the liner or prosthesis to facilitate manipulation with an upper limb prosthesis or the patient’s teeth. When initiating standing and ambulation training, adaptive crutches may be necessary, especially a “crutch prosthesis,” which essentially attaches a forearm crutch mechanism to an upper limb prosthetic socket. Additionally, assistive amputation training devices such as overhead suspension treadmills and Solostep^®^ systems can allow the patient to participate in mobility training, including walking, ramps, stairs, and running, while attached to a safety harness, enabling the patient and therapist the ability to work on proper gait training and symmetry in a safe environment.

## Wheelchairs, Special Adaptive Equipment, and Assistive Technology

Independent of the patient’s ability to ambulate with prosthetic devices, a proper wheelchair should be prescribed and fit for all individuals with bilateral lower limb amputation, even if only used for a backup for times of prosthetic malfunction. Not infrequently, skin complications such as blisters, ingrown hairs, folliculitis, or dermatitis will prohibit the use of a prosthesis, necessitating alternative mobility assistance with a wheelchair. Complications such as myodesis failure, heterotopic ossification, or symptomatic neuromata may necessitate surgical revision, resulting in protracted periods of time when prostheses may not be used [90]. A study examining the wheelchair use of individuals with traumatic limb loss from Vietnam and OIF/OEF, demonstrated that only 50.5 % of Vietnam and 42.8 % of OIF/OEF groups reported exclusive prosthetic use [91•]. More than half of individuals with lower limb loss reported using a wheelchair to assist with mobility even after prosthetic fitting, training, and years of prosthetic-assisted ambulation. The likelihood of wheelchair use was also shown to increase with bilateral lower limb amputation [91•]. Special considerations should be made during wheelchair set-up for an individual with multiple limb loss. Often a combination of powered and manual wheelchairs will be required for the patient with mixed upper and lower extremity limb loss. Use of a manual wheel chair can help augment cardiovascular activities while powered wheelchairs may be required for longer distances or times when the patient has upper extremity surgery, severe heterotrophic ossification, or upper extremity prosthetic failure. Special adaptations to upper extremity prosthetic devices are often required to propel manual wheelchairs for these individuals as well. Attaching specialty terminal devices to both body powered and myoelectric prostheses, as well as incorporating a rubberized surface to the posterior forearm section, can assist the individual in propelling their wheelchair. Special seating systems are necessary for those with hip disarticulation or hemipelvectomy. Individuals with bilateral above-knee amputations are best served with two wheelchairs: one to use with their prostheses and the other to be used without, as the configuration of the seating system and axis of the wheels will often need to be changed dramatically in these two situations.

While a full discussion of the various adaptive devices and assistive technologies available for individuals with multiple limb loss is beyond the scope of this manuscript, multiple options are now readily available to help enhance functional independence and successful community participation. Driving rehabilitation specialists can help individuals with a variety of impairments resume motor vehicle operation through training and adaptive equipment. Vocational and Assistive Technology specialists can provide those in need with computer aided devices, voice activated software, and universal control units that can greatly enhance inter-personal communication, household controls, and the pursuit of vocational aspirations. Finally, Sports and Recreational therapists can work closely with other therapists and prosthetists to develop creative solutions to allow resumption of virtually all sports and recreational pursuits, such as cycling (hand, recumbent, and upright), skiing (monoski, and conventional), golf, fishing, weight lifting, individual and team sports, creative arts, playing musical instruments, dancing, photography, and even world-class Paralympic competition. The reintegration into avocational activities cannot be underestimated. Patients partaking in avocational activities develop a sense of community and support with peers in similar rehabilitative situations and can engender a sense of independence and self-accomplishment. These changes can often be perceived as significant improvements in a patient’s demeanor and can contribute to decreases in reports of depressive symptoms as compared to before avocational engagement and improve patient participation in the rehabilitation process.

## Conclusion

Those with multiple limb loss face multitudes of challenges in their rehabilitation process, each of which is the result of the unique circumstance of that particular individual. Careful considerations are required to optimize the initial medical and surgical management, minimize behavioral health issues, and achieve proper pain control; all of which are necessary to maximize the chance of success of the rehabilitation process. Close multidisciplinary collaboration between the surgical team, medicine team, physiatrists, pain team, physical and occupational therapists, recreational and sports therapists, and prosthetists is essential to the success of the complex rehabilitation process. Social and family support is essential and can be maximized through both traditional and non-traditional channels such as support groups and peer visits. And finally, and perhaps most importantly, patient involvement from the very beginning of this process is absolutely necessary to identify realistic long-term goals for the patient, which are the guiding posts for the rehabilitation process.
